# Effect of Environmental Conditions on SARS-CoV-2 Stability in Human Nasal Mucus and Sputum

**DOI:** 10.3201/eid2609.202267

**Published:** 2020-09

**Authors:** M. Jeremiah Matson, Claude Kwe Yinda, Stephanie N. Seifert, Trenton Bushmaker, Robert J. Fischer, Neeltje van Doremalen, James O. Lloyd-Smith, Vincent J. Munster

**Affiliations:** Marshall University Joan C. Edwards School of Medicine, Huntington, West Virginia, USA (M.J. Matson);; Rocky Mountain Laboratories, National Institute of Allergy and Infectious Diseases, National Institutes of Health, Hamilton, Montana, USA (M.J. Matson, C. Kwe Yinda, S.N. Seifert, T. Bushmaker, R.J. Fischer, N. van Doremalen, V.J. Munster);; Montana State University, Bozeman, Montana, USA (T. Bushmaker);; University of California, Los Angeles, Los Angeles, California, USA (J.O. Lloyd-Smith)

**Keywords:** COVID-19, coronavirus disease, SARS-CoV-2, severe acute respiratory syndrome coronavirus 2, viruses, respiratory infections, zoonoses

## Abstract

We found that environmental conditions affect the stability of severe acute respiratory syndrome coronavirus 2 in nasal mucus and sputum. The virus is more stable at low-temperature and low-humidity conditions, whereas warmer temperature and higher humidity shortened half-life. Although infectious virus was undetectable after 48 hours, viral RNA remained detectable for 7 days.

Severe acute respiratory syndrome coronavirus 2 (SARS-CoV-2) is shed predominantly in upper and lower airway secretions (*1*), and transmission likely occurs predominantly through respiratory droplets, and potentially through direct contact and fomites. We describe SARS-CoV-2 stability in human nasal mucus and sputum under different environmental conditions.

We acquired pooled human nasal mucus and sputum commercially (Lee BioSolutions Inc., https://www.leebio.com) and mixed it with SARS-CoV-2 (SARS-CoV-2/human/USA/USA-WA1/2020) (*2*). We aliquoted 50 μL of each fluid containing 1 × 10^5^ 50% tissue culture infective dose/mL SARS-CoV-2 into sealed tubes (liquid setting) or onto polypropylene disks (surface setting), as previously described (*3*). We assessed stability under 3 environmental conditions: 4°C/40% relative humidity (RH), 21°C/40% RH, and 27°C/85% RH (RH applies only to exposed surface samples). We collected samples at specified timepoints and analyzed them for infectious virus by using endpoint titration. We extracted aliquots of collected surface samples by using the QIAGEN QIAamp Viral RNA Mini Kit (QIAGEN, https://www.qiagen.com) and analyzed them for the presence of viral RNA by using a quantitative reverse transcription PCR assay targeting the E gene (*4*). We fit linear regression models to log_10_-transformed titer data, calculated SARS-CoV-2 half-life (*t*_1/2_) for each condition, and tested differences by using analysis of covariance. We report all experimental measurements as means of 3 replicates with SE. We considered differences with p values <0.05 statistically significant.

We observed no significant differences in SARS-CoV-2 *t*_1/2_ between environmental conditions in liquid nasal mucus. In surface nasal mucus, SARS-CoV-2 *t*_1/2_ was significantly shorter at 27°C/85% RH compared with 21°C/40% RH (p = 0.0023) and 4°C/40% RH (p = 0.0007). At 27°C/85% RH, SARS-CoV-2 *t*_1/2_ also was significantly shorter in surface compared with liquid nasal mucus (p = 0.0101). Other comparisons of nasal mucus did not demonstrate significant differences in SARS-CoV-2 *t*_1/2_ (Table; Figure, panel A, B).

**Table:** Half-life (*t*_1/2_) for SARS-CoV-2 in human nasal mucus and sputum under different environmental conditions*

Sample and exposure type	Environment	Half-life, h (95% CI)
Nasal mucus		
Liquid	4°C	4.9 (3.5–8.7)
	21°C	3.7 (3.1–4.7)
	27°C	3.1 (2.3–4.4)
Surface	4°C/40% RH	3.3 (2.6–4.4)
	21°C/40% RH	3.1 (2.5–4.1)
	27°C/85% RH	1.5 (1.2–1.9)
Sputum		
Liquid	4°C	7.0 (5.8–8.9)
	21°C	1.9 (1.3–3.2)
	27°C	1.3 (1.1–1.7)
Surface	4°C/40% RH	5.8 (4.8–7.3)
	21°C/40% RH	3.1 (2.3–4.6)
	27°C/85% RH	1.5 (1.1–2.4)

*RH, relative humidity; SARS-CoV-2, severe acute respiratory syndrome coronavirus 2.

**Figure:**
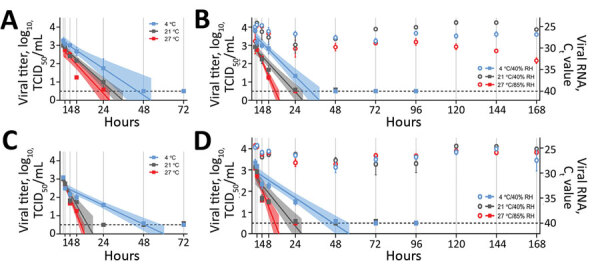
Stability of severe acute respiratory syndrome coronavirus 2 over time in human nasal mucus and sputum under different environmental conditions: liquid nasal mucus (A), surface nasal mucus (B), liquid sputum (C), and surface sputum (D). For panels B and D, the squares correspond to viral titer on the left y-axis, and the circles correspond to viral RNA (C_t_ value) on the right y-axis. We collected samples in 1 mL media for each condition at 0, 1, 4, 8, and 24 hours, then daily for 7 days and performed end-point titrations in quadruplicate on Vero E6 cells and made calculations using the Spearman-Kärber method. We log_10_-transformed and fit titers with linear regression models, including 95% CIs (shaded area around lines of best fit), by using GraphPad Prism 8 (https://www.graphpad.com). We extracted aliquots of collected surface samples by using the QIAamp Viral RNA Mini Kit (QIAGEN, https://www.qiagen.com) and analyzed them for the presence of viral RNA by using quantitative reverse transcription PCR targeting the E gene. For both viral titers and C_t_ values, plots show means of 3 replicates with SE. The limit of detection for each experimental condition was 10^0.5^ TCID_50_/mL for viral titer and 40 for C_t_ value and is indicated by the dashed line. Relative humidity is not applicable to liquid samples (panels A and C), which were in sealed tubes. C_t_, cycle threshold; RH, relative humidity; TCID_50_/mL, 50% tissue culture infective dose/mL.

SARS-CoV-2 *t*_1/2_ was significantly longer in liquid sputum at 4°C than at 21°C (p = 0.0006) and 27°C (p<0.0001). In surface sputum, SARS-CoV-2 *t*_1/2_ also was significantly longer at 4°C/40% RH than at 21°C/40% RH (p = 0.0042) and 27°C/85% RH (p = 0.0002). In addition, SARS-CoV-2 *t*_1/2_ was significantly longer at 21°C/40% RH than 27°C/85% RH (p = 0.0027) in surface sputum. We observed no significant differences in SARS-CoV-2 *t*_1/2_ between liquid and surface sputum (Table; Figure, panel C, D). SARS-CoV-2 RNA remained detectable for >7 days in all surface samples, with a gradual increase in cycle threshold value (decrease in detected RNA) occurring only in nasal mucus at 27°C/85% RH (Figure, panel B, D).

We previously reported on the surface stability of SARS-CoV-2 in culture media at 21°C/40% RH (*3*). However, SARS-CoV-2 stability is affected by its surrounding matrix and environmental conditions. The *t*_1/2_ we report here for SARS-CoV-2 in surface nasal mucus and sputum at 21°C/40% (Table) is considerably shorter than what we found in culture media under similar conditions (*t*_1/2_ 6.8 [95% CI 5.6–8.2] hours) (*3*). In addition, we set out to determine SARS-CoV-2 stability in nasal mucus and sputum under environmental conditions that approximate temperate winter (4°C/40% RH), climate-controlled (21°C/40% RH), and temperate summer or tropical (27°C/85% RH) settings. SARS-CoV-2 was generally more stable at cooler temperatures and lower RH, and less stable at warmer temperatures and higher RH. Nevertheless, with our experimental protocol and initial titer, we predicted that SARS-CoV-2 would remain infectious in nasal mucus and sputum on surfaces for >10–12 hours even in warm, humid conditions. However, the amount of infectious SARS-CoV-2 shed and virus stability in relationship to infectious dose for humans are currently unknown, hampering conclusions regarding infectious duration and transmission. The general similarity in SARS-CoV-2 stability between liquid and surface samples suggests that in general temperature factored more heavily than humidity.

Community transmission of SARS-CoV-2 is widespread (*5*) and might be explained by contact with asymptomatic or presymptomatic (*6*) infected persons. Because of the surface stability of SARS-CoV-2, fomite transmission might also play a role. In addition, reduced surface stability of SARS-CoV-2 in human nasal mucus and sputum in warmer and more humid conditions might result in decreased virus transmission, and climatic influence on SARS-CoV-2 transmission rates might eventually drive seasonal outbreak dynamics in a postpandemic period (*7*), similar to other respiratory viruses (e.g., influenza A virus or human coronavirus OC43).

SARS-CoV-2 RNA has been detected on surfaces throughout clinical settings (*8*,*9*) and aboard a cruise ship for extended periods (*10*), but any correlation to infectious virus was previously unknown. In our study, infectious virus persisted in both nasal mucus and sputum on surfaces for ≈24 hours under climate-controlled conditions. However, viral RNA was consistently detectable for >7 days under various conditions in both nasal mucus and sputum on surfaces. These findings suggest that inferences regarding the presence of infectious virus from quantitative reverse transcription PCR data alone should be made with caution.

## References

[1] Zhu N, Zhang D, Wang W, Li X, Yang B, Song J, et al.; China Novel Coronavirus Investigating and Research Team. A novel coronavirus from patients with pneumonia in China, 2019. N Engl J Med. 2020;382:727–33. 10.1056/NEJMoa2001017 31978945 PMC7092803

[2] Harcourt J, Tamin A, Lu X, Kamili S, Sakthivel SK, Murray J, et al. Severe acute respiratory syndrome coronavirus 2 from patient with coronavirus disease, United States. Emerg Infect Dis. 2020;26:1266–73. 10.3201/eid2606.200516 32160149 PMC7258473

[3] van Doremalen N, Bushmaker T, Morris DH, Holbrook MG, Gamble A, Williamson BN, et al. Aerosol and surface stability of SARS-CoV-2 as compared with SARS-CoV-1. N Engl J Med. 2020;382:1564–7. 10.1056/NEJMc2004973 32182409 PMC7121658

[4] Corman VM, Landt O, Kaiser M, Molenkamp R, Meijer A, Chu DK, et al. Detection of 2019 novel coronavirus (2019-nCoV) by real-time RT-PCR. Euro Surveill. 2020;25:25. 10.2807/1560-7917.ES.2020.25.3.2000045 31992387 PMC6988269

[5] Liu J, Liao X, Qian S, Yuan J, Wang F, Liu Y, et al. Community transmission of severe acute respiratory syndrome coronavirus 2, Shenzhen, China, 2020. Emerg Infect Dis. 2020;26:1320–3. 10.3201/eid2606.200239 32125269 PMC7258448

[6] He X, Lau EHY, Wu P, Deng X, Wang J, Hao X, et al. Temporal dynamics in viral shedding and transmissibility of COVID-19. Nat Med. 2020;26:672–5. 10.1038/s41591-020-0869-5 32296168

[7] Kissler SM, Tedijanto C, Goldstein E, Grad YH, Lipsitch M. Projecting the transmission dynamics of SARS-CoV-2 through the postpandemic period. Science. 2020;368:860–8. 10.1126/science.abb5793 32291278 PMC7164482

[8] Ong SWX, Tan YK, Chia PY, Lee TH, Ng OT, Wong MSY, et al. Air, surface environmental, and personal protective equipment contamination by severe acute respiratory syndrome coronavirus 2 (SARS-CoV-2) from a symptomatic patient. JAMA. 2020;323:1610. 10.1001/jama.2020.3227 32129805 PMC7057172

[9] Guo ZD, Wang ZY, Zhang SF, Li X, Li L, Li C, et al. Aerosol and surface distribution of severe acute respiratory syndrome coronavirus 2 in hospital wards, Wuhan, China, 2020. Emerg Infect Dis. 2020;26:26. 10.3201/eid2607.200885 32275497 PMC7323510

[10] Moriarty LF, Plucinski MM, Marston BJ, Kurbatova EV, Knust B, Murray EL, et al.; CDC Cruise Ship Response Team; California Department of Public Health COVID-19 Team; Solano County COVID-19 Team. California Department of Public Health COVID-19 Team; Solano County COVID-19 Team. Public health responses to COVID-19 outbreaks on cruise ships—worldwide, February–March 2020. MMWR Morb Mortal Wkly Rep. 2020;69:347–52. 10.15585/mmwr.mm6912e3 32214086 PMC7725517

